# The first report of structural analysis of a nucleic acid using crystals grown in space

**DOI:** 10.1107/S2053230X25000810

**Published:** 2025-02-12

**Authors:** Shin Ando, Moena Takahashi, Jiro Kondo

**Affiliations:** a Graduate School of Science and Technology Sophia University 7-1 Kioi-cho, Chiyoda-ku Tokyo102-8554 Japan; b Department of Materials and Life Sciences, Faculty of Science and Technology Sophia University 7-1 Kioi-cho, Chiyoda-ku Tokyo102-8554 Japan; Osaka University, Japan

**Keywords:** X-ray crystallography, nucleic acids, microgravity

## Abstract

For the success of structure-based drug design, three-dimensional structures solved by X-ray crystallography at atomic resolution are mandatory. In order to obtain high-quality single crystals with strong diffraction power, crystallization under microgravity conditions has been attempted for proteins. Since nucleic acid duplexes have chemical, structural and crystallographic characteristics that differ from those of globular proteins, such as intermolecular repulsion due to negative charge and molecular and crystallographic anisotropies, it is interesting to investigate whether microgravity crystallization improves the crystal growth of nucleic acids. However, to our knowledge there has been only one report on nucleic acid crystallization in a microgravity environment, and there have been no reports of successful structural analysis. Here, we conducted the crystallization of a DNA/RNA heteroduplex in space. The heteroduplex was successfully crystallized in a microgravity environment, and the size and appearance of the crystals were improved compared with control experiments conducted on Earth. Although the effect of the counter-diffusion method is likely to be more significant than the effect of microgravity in this study, we were able to analyze the structure at a higher resolution (1.4 Å) than our previously reported crystal structure (1.9 Å).

## Abstract 2

The first structural analysis of a nucleic acid using crystals obtained under microgravity crystallization in space is reported. A DNA/RNA heteroduplex was successfully crystallized in a microgravity environment, improving the size and appearance of the crystals compared with crystals obtained on Earth.

## 1. Introduction

With new modalities on the rise, nucleic acids are attracting attention as targets for conventional small-molecule drugs as well as as nucleic acid therapeutics in their own right (Belgrad *et al.*, 2024 ▸; Childs-Disney *et al.*, 2022 ▸). In both cases, structure-based drug design (SBDD) is one of the rational and promising strategies to develop medications with high affinity and specificity (high efficacy and lower toxicity). Therefore, there is an increasing need to observe 3D structures of nucleic acids at atomic resolution using X-ray crystallography (Egli & Manoharan, 2023 ▸; Kondo, 2016 ▸). This method requires high-quality single crystals with few lattice defects and high diffraction performance.

Crystallization is initiated by adding precipitants to a homogeneously distributed solution of biomacromolecules, which causes the molecules to associate with each other and form crystal nuclei. These nuclei then grow by incorporating surrounding molecules. At this stage, the concentration of the solution around the crystal is lower than in areas further away from the nuclei, resulting in a density gradient that causes the low-concentration solution to move vertically upwards against gravity (Pusey *et al.*, 1988 ▸). Consequently, higher concentration solutions flow in, leading to rapid changes in the concentration near the crystal surface and varying concentrations across different crystal faces. Thus, when crystals are formed on Earth, their quality may deteriorate because the convection currents caused by density differences due to gravity may cause new crystal growth on the crystal surface and/or prevent regular crystal growth. On the other hand, in a microgravity environment convection due to density differences is minimal and biomolecules move only by diffusion, which maintains a low-concentration area around the crystal nuclei, allowing slow and stable crystal growth and improving the crystal quality (McPherson, 1999 ▸).

Therefore, attempts have been made to crystallize several proteins in space, and improvements in resolution have been observed which can be utilized for structural analysis (McPherson & DeLucas, 2015 ▸). To the best of our knowledge, there is only one report of nucleic acid crystallization in a microgravity environment, and the results did not have sufficient resolution to be used for structural analysis or investigation of the effects of gravity (Lorenz *et al.*, 2000 ▸). Since nucleic acids have characteristic double-helical structures, they tend to stack at the end of the molecules to pack along the helical axis (Shamim *et al.*, 2020 ▸), thereby forming anisotropic crystals. As a result, X-ray diffraction spots only appear in certain areas. In addition, nucleic acids are entirely covered with negative charges derived from phosphate groups, whereas in proteins positive and negative charges are distributed throughout the molecule. Therefore, the solubility curve of nucleic acids generally shows different characteristics to those of proteins, and the crystallization conditions are also significantly different. For nucleic acids with these characteristics, it is imperative to examine in detail whether crystallization under microgravity conditions effectively improves the quality of crystals and how it impacts structural analysis. In this study, we conducted the crystallization of a DNA/RNA heteroduplex in space and provide the first report on nucleic acid crystallization under microgravity and subsequent structure analyses.

## 2. Materials and methods

### 2.1. Sample preparation and crystallization

Based on previous studies (Xiong & Sundaralingam, 2000 ▸; Kondo *et al.*, 2016 ▸), a nonameric DNA with the sequence d(*X*TCTTCTT*X*) (where *X* is 5-methyl-dC) and a nonameric RNA with the sequence r(GAAGAAGAG) were chemically synthesized by an automated nucleic acid synthesizer and were purified by HPLC and reversed-phase column chromatography.

Crystallizations were carried out on the Japanese Experiment Module ‘Kibo’ of the International Space Station (ISS) after verification of conditions on Earth. Both on Earth and in space, crystallizations were performed under two different conditions using the counter-diffusion method in a container called JCB-SGT (Takahashi *et al.*, 2019 ▸).

For the first condition, a capillary containing 25 µl nucleic acid sample solution (12.5 µl 4 m*M* DNA solution and 12.5 µl 4 m*M* RNA solution) and 25 µl crystallization solution [150 m*M* NaCl, 20% 2-methyl-2,4-pentanediol (MPD), 10 m*M* spermine·4HCl, 50 m*M* 2-(*N*-morpholino)ethanesulfonic acid (MES) pH 6.5] was connected to one end of a silicone tube filled with 1 mm diameter × 12 mm 1%(*w*/*v*) agarose gel and the other end was inserted into ∼1200 µl reservoir solution (150 m*M* NaCl, 40% MPD, 10 m*M* spermine·4HCl, 50 m*M* MES pH 6.5) and left to stand.

For the second condition, a capillary containing 25 µl nucleic acid sample solution (12.5 µl 2 m*M* DNA solution and 12.5 µl 2 m*M* RNA solution) and 25 µl crystallization solution (10 m*M* KCl, 10% MPD, 10 m*M* spermine, 50 m*M* MES pH 6.5) was connected to one end of a silicone tube filled with agarose gel and the other end was inserted into reservoir solution (10 m*M* KCl, 40% MPD, 10 m*M* spermine, 50 m*M* MES pH 6.5) and left to stand.

These crystallization conditions are summarized in Table 1 ▸. In all conditions, the concentrations of the crystallization solution and the reservoir solution were set to be equal except for the MPD concentration. This is because the concentrations of additives other than MPD do not change when the solution flows in and out through the gel, whereas the concentration of the precipitant MPD increases from its initial concentration in the capillary to that of the reservoir solution, causing crystal nucleation and subsequent crystal growth.

### 2.2. X-ray data collection

All X-ray diffraction data were obtained using synchrotron radiation at 100 K.

A diffraction data set was collected from a crystal obtained under the first crystallization condition on Earth (hereafter referred to as the Earth-1 crystal) on BL41XU at SPring-8. An X-ray beam with a wavelength of 1.00 Å was used and the diffraction data were measured with 0.2° oscillation and 0.2 s exposure time per image on a pixel detector (EIGER X 16M) placed 190 mm away from the crystal. A data set was also collected from a crystal grown under the first condition in space (hereafter referred to as the the Space-1 crystal) on BL41XU at SPring-8. The wavelength of the X-ray beam was 0.80 Å and the diffraction patterns were recorded using an EIGER X 16M detector positioned 180 mm away from the crystal by oscillating the goniometer in 0.2° increments with 0.20 s exposure per frame.

Diffraction data sets were collected from a crystal obtained under the second crystallization condition both on Earth and in space (Earth-2 crystal and Space-2 crystal, respectively) on BL17A at Photon Factory (PF). Using an X-ray beam with a wavelength of 0.98 Å, diffraction images were collected on an EIGER X 16M detector, which was positioned 70 mm away from the crystal, with 1° oscillation and 1 s exposure time per frame.

These measurement conditions are also summarized in Table 2 ▸.

All X-ray data were processed using *XDS* (Kabsch, 2010 ▸). The data-collection statistics and crystal data are summarized in Table 3 ▸.

### 2.3. Structure determination and refinement

The initial phases were determined by the molecular-replacement method using *Phaser-MR* from the *Phenix* suite (Liebschner *et al.*, 2019 ▸; McCoy *et al.*, 2007 ▸). The molecular structures were manipulated in *Coot* (Emsley *et al.*, 2010 ▸). The atomic parameters of each structure were refined using *phenix.refine* from the *Phenix* suite (Afonine *et al.*, 2012 ▸; Liebschner *et al.*, 2019 ▸) through a combination of simulated annealing, crystallographic conjugate-gradient minimization refinement and *B*-factor refinement. The structure-refinement statistics are summarized in Table 3 ▸.

The structures of the Earth-1, Space-1, Earth-2 and Space-2 crystals were deposited in the Protein Data Bank (PDB) with accession codes 9kkz, 9kl1, 9k7r and 9k8z, respectively.

## 3. Results and discussion

### 3.1. Effects of microgravity crystallization on the size, appearance and quality of crystals

**Figure 1:**
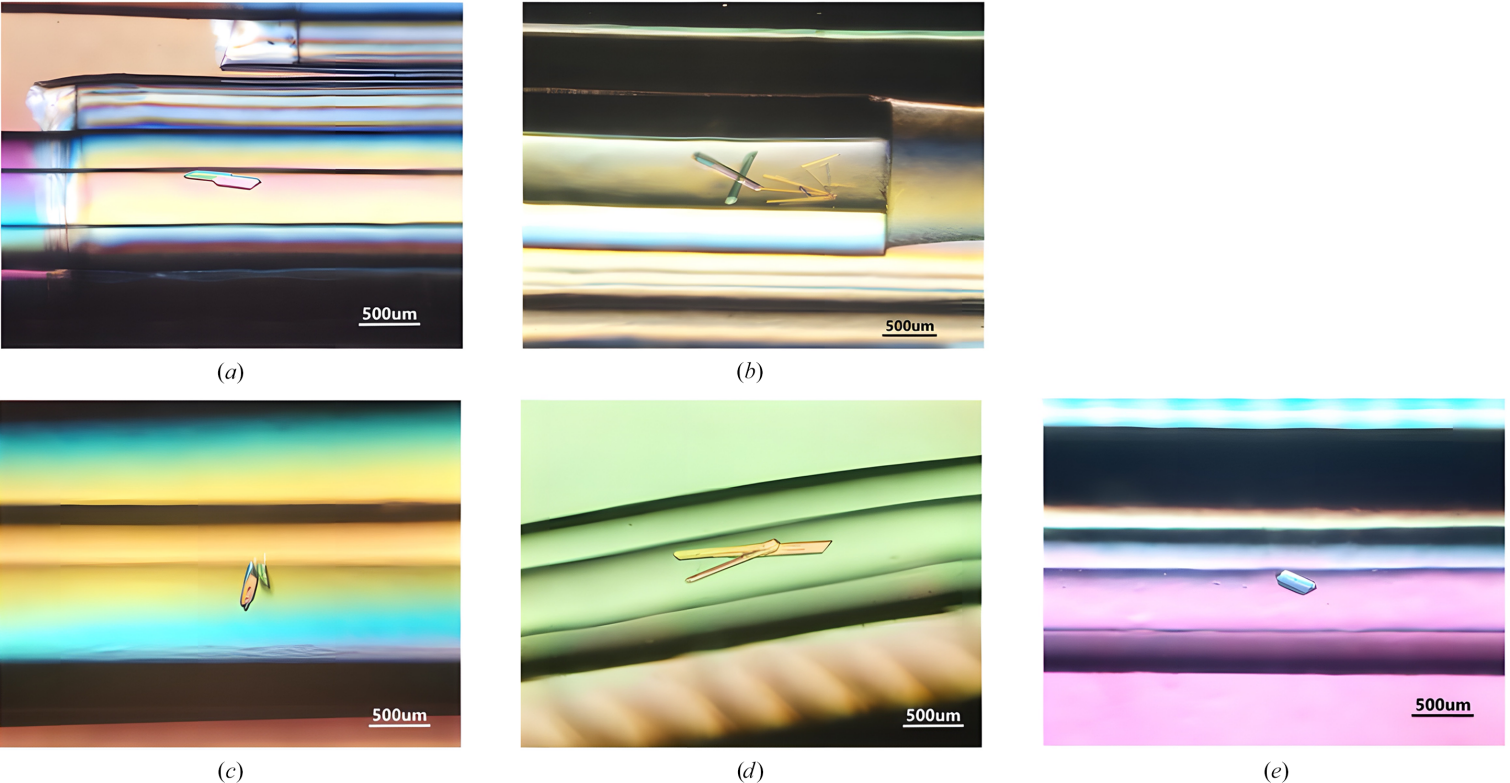
(*a*) Earth-1 crystal. (*b*) Space-1 crystals. (*c*) Earth-2 crystals. (*d*) Space-2 polycrystal. (*e*) Space-2 single crystal.

Under the first crystallization condition, crystallization on Earth performed as a control experiment resulted in columnar single crystals with a maximum major axis of about 400 µm (Fig. 1 ▸*a*). On the other hand, crystallization in space resulted in columnar single crystals with a maximum major axis of about 550 µm (Fig. 1 ▸*b*).

Under the second crystallization condition, crystallization on Earth resulted in columnar single crystals (Fig. 1 ▸*c*) and a columnar polycrystal (Fig. 1 ▸*d*). The lengths of the largest single crystal and the polycrystal were about 330 and 1260 µm, respectively. On the other hand, crystallization in space resulted in only a single columnar crystal (Fig. 1 ▸*e*). The length of the largest crystal was about 360 µm.

The crystal data, the data-collection statistics and the structure-refinement statistics are summarized in Table 3 ▸.

All of the crystals obtained both on Earth and in space belonged to space group *P*6_1_, with almost identical unit-cell dimensions. They are also isomorphous with the crystal obtained using the hanging-drop vapor-diffusion method in our previous study (Kondo *et al.*, 2016 ▸). In brief, it was revealed that microgravity does not affect the packing of nucleic acid molecules.

The maximum resolution determined according to the same criteria (the completeness is >99%; the *R*_merge_ of the outer shell is around 35% or less; the multiplicity is about 5; the *I*/σ(*I*) in the outer shell is >3; the CC_1/2_ in the outer shell is >60%) was 1.5 Å for the Earth-1 crystal, 1.6 Å for the Space-1 crystal, 1.6 Å for the Earth-2 crystal and 1.4 Å for the Space-2 crystal. The results showed no significant difference in the maximum resolution and crystal mosaicity between Earth and a microgravity environment. However, the maximum resolution significantly improved compared with our previous study (Kondo *et al.*, 2016 ▸), where the crystal structure was solved at a maximum resolution of 1.9 Å. In the previous study, crystallization was conducted using the hanging-drop vapor-diffusion method, whereas in this study crystallization was performed using the counter-diffusion method. Therefore, the effect of the counter-diffusion method is likely to be more significant than the effect of microgravity in this study.

As this was our first attempt at microgravity crystallization of nucleic acids, we chose the DNA/RNA heteroduplex as a promising sample to crystallize. As we expected, we succeeded in crystallizing this sample in space, and we were able to investigate the effect of a microgravity environment on the size and appearance of the crystals. However, since this sample tends to crystallize with good quality and strong diffraction power, it was difficult to investigate the effects of microgravity on resolution and data quality. We are currently attempting to increase the amount of data to make the data analysis more effective.

### 3.2. Effect of microgravity crystallization on the refined structure

**Figure 2:**
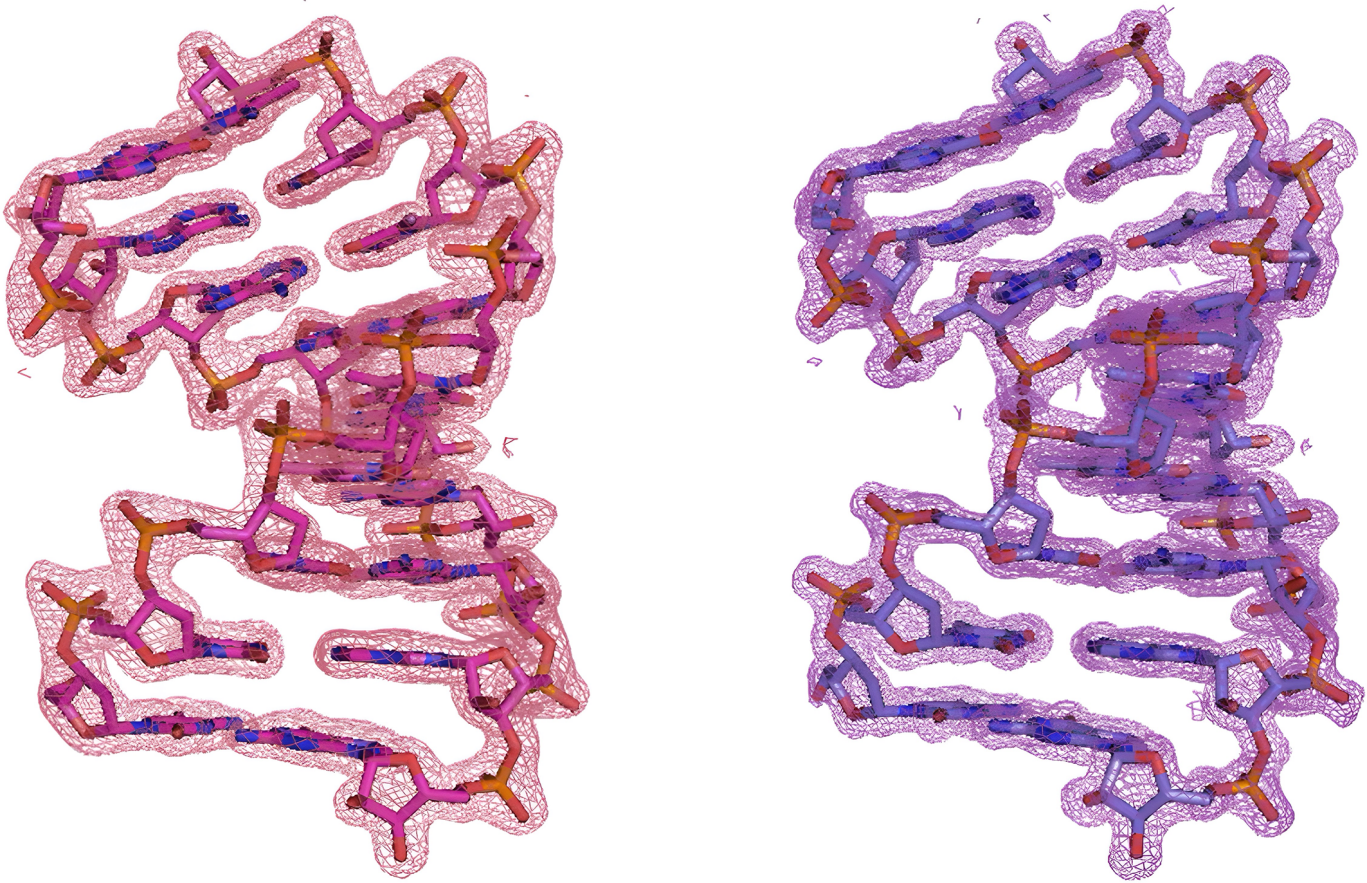
Overall structures of DNA/RNA duplexes obtained in the previous study (left) and in the second condition in space (right). The 2*mF*_o_ − *DF*_c_ electron density is contoured at a 2.0σ level. Ligands and water molecules are omitted.

**Figure 3:**
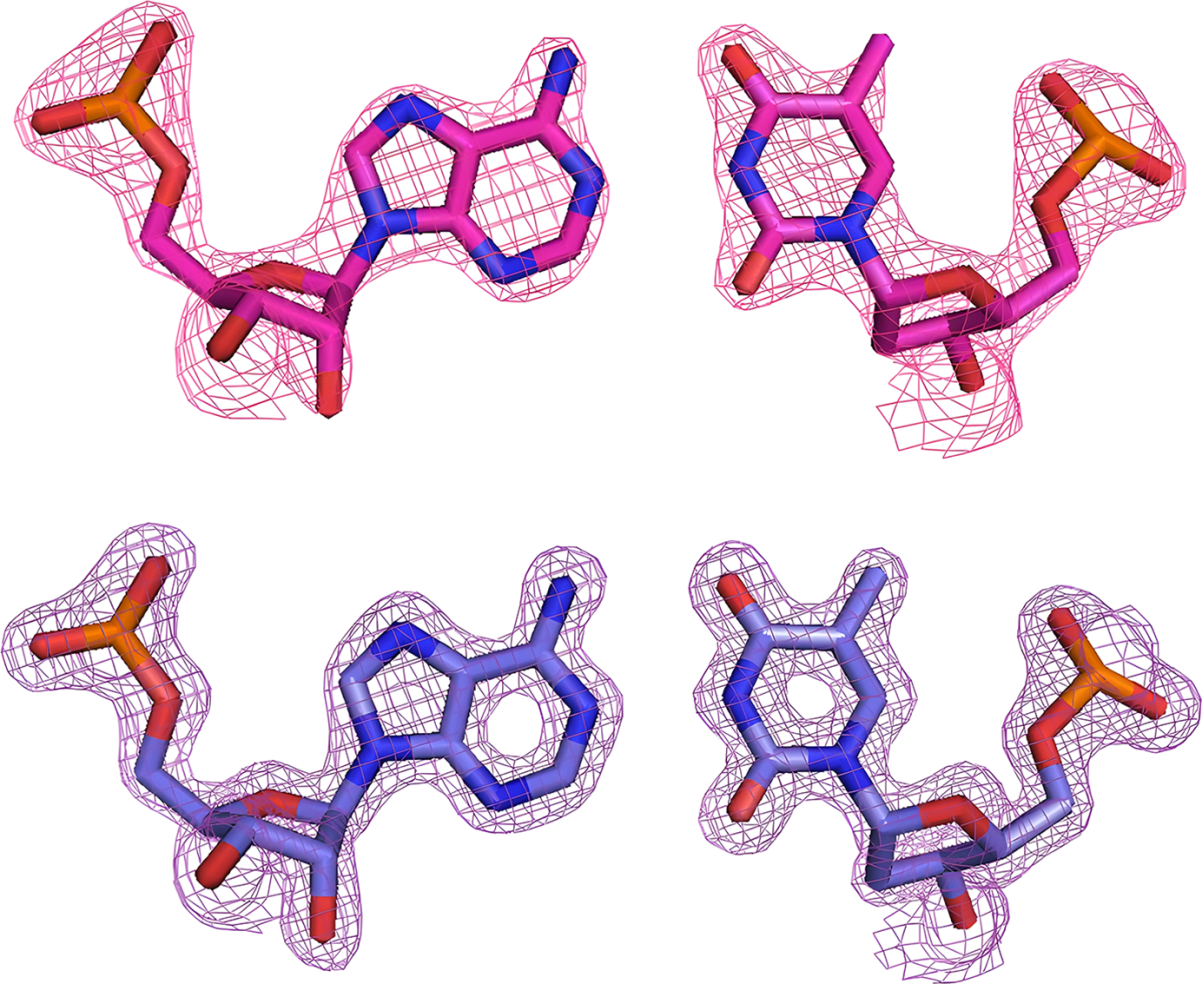
A Watson–Crick A–T base pair observed in the DNA/RNA duplexes obtained in the previous study (top) and in the second condition in space (bottom). The 2*mF*_o_ − *DF*_c_ electron densities are contoured at a 2.0σ level.

The refined three-dimensional structures are shown in Fig. 2 ▸ and Supplementary Fig. S1. All structures are similar to that determined in the previous study (Kondo *et al.*, 2016 ▸). When the previously solved structure (PDB entry 4u6m) was superimposed with the structures determined in this study, the root-mean-square deviations (r.m.s.d.s) for all atoms were less than 0.2 Å (0.116 Å with the Earth-1 structure, 0.139 Å with the Space-1 structure, 0.161 Å with the Earth-2 structure and 0.172 Å with the Space-2 structure). On the other hand, as mentioned above, the structure determined in this study had an improved maximum resolution compared with our previous study, and therefore the 2*mF*_o_ − *DF*_c_ electron-density map clearly improved (Fig. 3 ▸). There was no significant difference in the average *B*-factor values of the DNA/RNA double helix depending on whether crystallization was performed on Earth or in space, but there was a tendency for these to depend on the resolution (Table 3 ▸). Overall structures colored to represent the *B* factor are shown in Supplementary Fig. S3.

Analysis of the helical parameters using 3*DNA* (Li *et al.*, 2019 ▸), shown in Supplementary Fig. S2, shows that all parameters are fairly consistent among all of the structures.

A comparison of the number of water molecules observed shows no notable differences: 97 in the previous study (Kondo *et al.*, 2016 ▸), 66 in the Earth-1 crystal, 53 in the Space-1 crystal, 76 in the Earth-2 crystal and 82 in the Space-2 crystal. Incidentally, spermine was observed due to the improved resolution in all four refined structures.

As expected from the lack of effect on the lattice constants and space group, gravity had almost no effect on the three-dimensional structure of RNA/DNA. It is generally known that the helical structure of nucleic acids changes depending on sugar puckering, helical parameters, hydration and so on. In this study, no artificial structural disruptions were observed in a microgravity environment, suggesting that microgravity crystallization is also applicable to nucleic acids.

## 4. Conclusion

In this study, we succeeded in structural analysis of a nucleic acid using crystals obtained under a microgravity environment. We confirmed that microgravity crystallization affects the size and appearance of the crystals, but has little effect on the diffraction data quality. In this study, the effects of microgravity were limited, but as is the case with proteins, the effects could be different depending on the structure and sequence of the nucleic acid and the space group of the crystal. Through this experiment, we were able to establish a methodology for microgravity crystallization of nucleic acids. In the next step, we would like to expand the scope of our research to include various types of nucleic acids, such as bulges, internal loops, hairpin loops, triplexes and quadruplexes, to contribute to the development of the structure-based design of nucleic acid-targeted drugs and nucleic acid therapeutics.

## Supplementary Material

PDB reference: DNA/RNA heteroduplex, K form, crystals grown on Earth, 9k7r

PDB reference: crystals grown in space, 9k8z

PDB reference: Na form, crystals grown on Earth, 9kkz

PDB reference: crystals grown in space, 9kl1

Supplementary Figures. DOI: 10.1107/S2053230X25000810/nw5129sup1.pdf

## Figures and Tables

**Table 1:** Crystallization conditions

Crystal	Earth-1	Space-1	Earth-2	Space-2
PDB code	9kkz	9kl1	9k7r	9k8z
Sample solution (25 µl)				
DNA (m*M*)	2	2	1	1
RNA (m*M*)	2	2	1	1
Crystallization solution (25 µl)				
MES pH 6.5 (m*M*)	50	50	50	50
NaCl (m*M*)	150	150	—	—
KCl (m*M*)	—	—	10	10
Spermine (m*M*)	—	—	10	10
Spermine·4HCl (m*M*)	10	10	—	—
MPD (%)	20	20	10	10
Reservoir solution (∼1200 µl)				
MES pH 6.5	50	50	50	50
NaCl (m*M*)	150	150	—	—
KCl (m*M*)	—	—	10	10
Spermine (m*M*)	—	—	10	10
Spermine·4HCl (m*M*)	10	10	—	—
MPD (%)	40	40	40	40

**Table 2:** Conditions of X-ray diffraction experiments

Crystal	Earth-1	Space-1	Earth-2	Space-2
PDB code	9kkz	9kl1	9k7r	9k8z
Beamline	BL41XU, SPring-8	BL41XU, SPring-8	BL17A, PF	BL17A, PF
Wavelength (Å)	1.0	0.80	0.98	0.98
Beam size (µm)	10 × 10	10 × 10	40 × 20	40 × 20
Exposure time per image (s)	0.2	0.2	1	1
Oscillation range per image (°)	0.2	0.2	1	1
Total No. of images	90	90	90	90
Distance to the detector (mm)	190	180	70	70

**Table 3:** Crystal data, data-collection statistics and structure-refinement statistics Values in parentheses are for the outer shell.

Crystal	Previous study	Earth-1	Space-1	Earth-2	Space-2
PDB code	4u6m	9kkz	9kl1	9k7r	9k8z
Crystal data					
Space group	*P*6_1_	*P*6_1_	*P*6_1_	*P*6_1_	*P*6_1_
*a*, *b*, *c* (Å)	49.1, 49.1, 46.0	48.8, 48.8, 45.8	49.0, 49.0, 45.7	48.5, 48.5, 45.6	48.7, 48.7, 45.7
No. of NA strands in ASU†	1	1	1	1	1
Data collection					
Beamline	BL5A, PF	BL41XU, SPring-8	BL41XU, SPring-8	BL17A, PF	BL17A, PF
Wavelength (Å)	1.0	1.0	0.80	0.98	0.98
Resolution (Å)	42.5–1.9 (2.0–1.9)	31.1–1.5 (1.6–1.5)	31.1–1.6 (1.7–1.6)	24.2–1.6 (1.7–1.6)	24.3–1.4 (1.5–1.4)
Unique reflections	5022	10053	8323	8199	12207
Completeness (%)	99.8 (99.8)	99.9 (100.0)	99.7 (99.3)	99.9 (99.9)	99.9 (100.0)
*R*_merge_‡ (%)	4.0 (35.8)	2.9 (25.6)	2.7 (32.8)	3.5 (38.6)	3.8 (35.1)
*R*_meas_§ (%)	6.4 (37.4)	2.9 (25.6)	3.0 (36.5)	3.9 (43.3)	4.2 (38.6)
Multiplicity	10.2 (10.3)	5.0 (5.1)	5.2 (5.1)	5.1 (4.9)	5.5 (5.9)
〈*I*/σ(*I*)〉	23.2 (5.7)	27.8 (6.2)	26.0 (4.6)	22.3 (4.0)	20.7 (3.8)
CC_1/2_		99.9 (97.1)	100.0 (94.9)	100.0 (92.4)	99.9 (93.0)
Mosaicity (°)		0.226	0.179	0.275	0.393
Structure refinement					
Resolution range (Å)	42.5–1.9	31.1–1.5	31.1–1.6	24.2–1.6	24.3–1.4
Reflections used	5020	10053	8323	8199	12207
*R* factor¶ (%)	20.7	19.3	19.6	18.1	18.5
*R*_free_†† (%)	23.3	21.1	20.9	19.6	19.6
R.m.s.d., bond lengths (Å)	0.006	0.007	0.006	0.008	0.006
R.m.s.d., bond angles (°)	1.2	1.1	1.0	1.0	1.0
Average *B* factor (Å^2^)	35.6	26.9	27.7	26.1	22.2

† Number of nucleic acid strands in the asymmetric unit.

‡ *R*_merge_ = 100 × 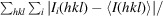

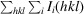
.

§ *R*_meas_ = 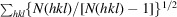

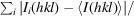

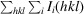
.

¶ *R* factor = 100 × 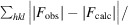

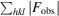
, where |*F*_obs_| and |*F*_calc_| are optimally scaled observed and calculated structure-factor amplitudes, respectively.

†† Calculated using a random set containing 10% of observations.
