# The first report of structural analysis of a nucleic acid using crystals grown in space. Corrigendum

**DOI:** 10.1107/S2053230X25002766

**Published:** 2025-04-01

**Authors:** Shin Ando, Moena Takahashi, Jiro Kondo

**Affiliations:** a Graduate School of Science and Technology Sophia University 7-1 Kioi-cho, Chiyoda-ku Tokyo102-8554 Japan; b Department of Materials and Life Sciences, Faculty of Science and Technology Sophia University 7-1 Kioi-cho, Chiyoda-ku Tokyo102-8554 Japan

**Keywords:** X-ray crystallography, nucleic acids, microgravity

## Abstract

Corrections are made to the article by Ando *et al.*[(2025), *Acta Cryst.* F**81**, 95–100].

## Abstract 2

The article by Ando *et al.*[(2025), *Acta Cryst.* F**81**, 95–100] is corrected.

In the *Introduction* of the article by Ando *et al.* (2025 ▸; page 96, lines 12–16 of the left column), we previously wrote:

To the best of our knowledge, there is only one report of nucleic acid crystallization in a microgravity environment, and the results did not have sufficient resolution to be used for structural analysis or investigation of the effects of gravity (Lorenz *et al.*, 2000 ▸).

However, we have realised that this statement was inaccurate. In fact, several significant studies have successfully achieved nucleic acid crystallization in microgravity environments, followed by structural determination.

The corrected sentence should read as follows:

Several studies have reported nucleic acid crystallization and subsequent structure determination in a microgravity environment (Lorenz *et al.*, 2000 ▸; Vallazza *et al.*, 2002 ▸, 2004 ▸; Rypniewski *et al.*, 2006 ▸). These valuable studies have shown that microgravity can positively influence crystal quality. However, systematic evaluations of the impact of microgravity on crystallization and structural features remain limited.

Additionally, at the end of the *Introduction*, we previously wrote:

In this study, we conducted the crystallization of a DNA/RNA heteroduplex in space and provide the first report on nucleic acid crystallization under microgravity and subsequent structure analyses.

However, this statement also contained an inaccuracy, as previous studies have reported RNA crystallization under microgravity and structural determination.

The corrected sentence should read as follows:

In this study, we conducted the crystallization of a DNA/RNA heteroduplex in space and present a report of systematic evaluation of nucleic acid crystallization under microgravity, providing new perspectives on how microgravity conditions may influence crystal quality and structural analyses.

We also acknowledge that the title of our paper may not fully reflect the historical context of microgravity nucleic acid crystallization studies, and we apologize for any confusion that may have arisen as a result.

This correction does not affect the overall conclusions of our study.
