# Supplementary material for: The first report of structural analysis of a nucleic acid using crystals grown in space
Source: Acta Crystallogr F Struct Biol Commun. 2025 Feb 12;81(Pt 3):95–100. doi: 10.1107/S2053230X25000810 (PMC11866410; doi:10.1107/S2053230X25000810)
Supplement: Supplementary file 1 [file f-81-00095-sup1.pdf]

STRUCTURAL BIOLOGY
COMMUNICATIONS

Volume 81 (2025)

Supporting information for article:

The first report of structural analysis of a nucleic acid using crystals grown in space

Shin Ando, Moena Takahashi and Jiro Kondo

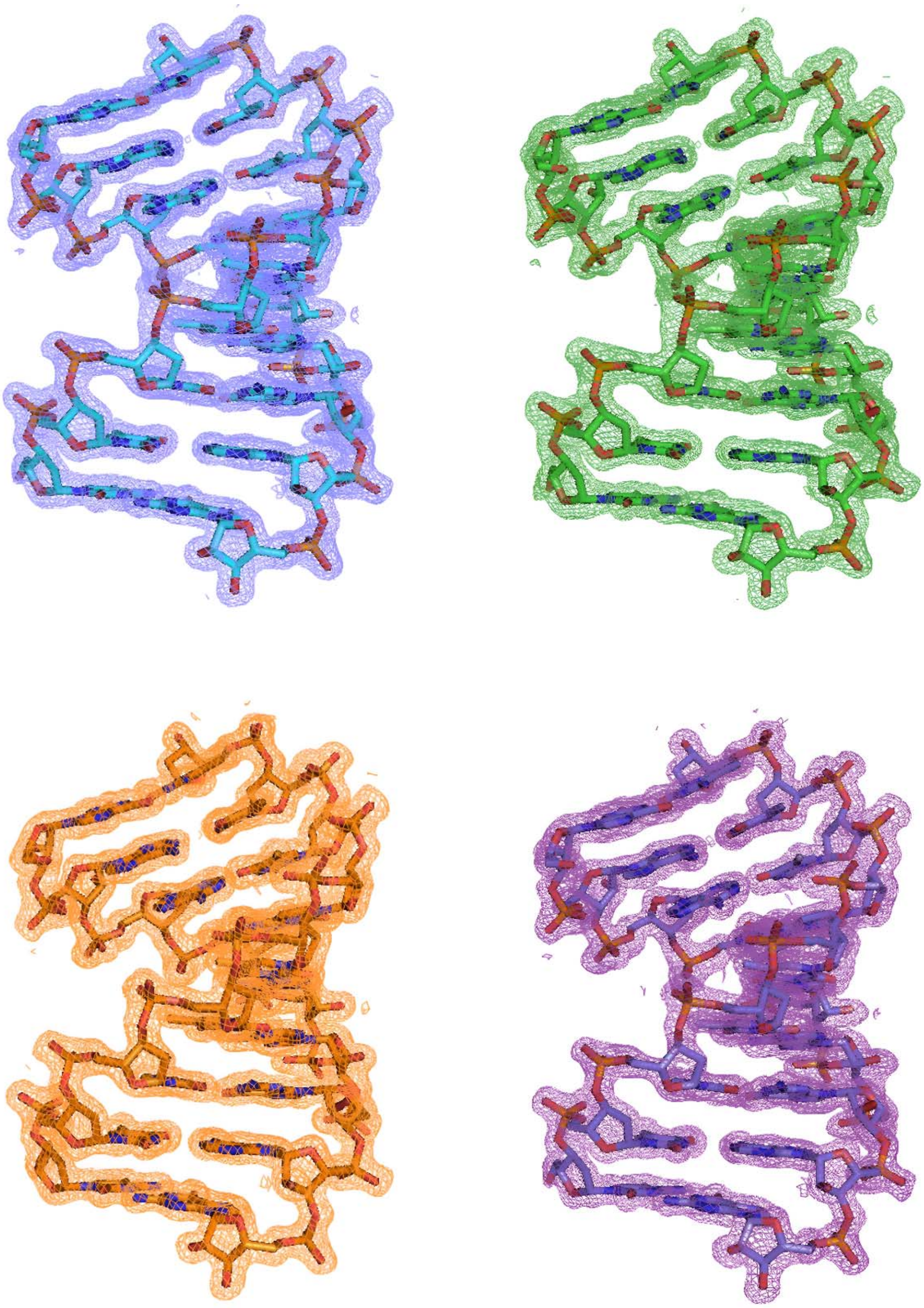

Figure S1 Overall structures of DNA/RNA duplexes obtained in the Earth-1 (blue), in the Space-1 (green), in the Earth-2 (orange), and in the Space-2 (violet). The 2mFo-DFc electron density is contoured at a 2.0 sigma level. Ligands and water molecules are omitted.

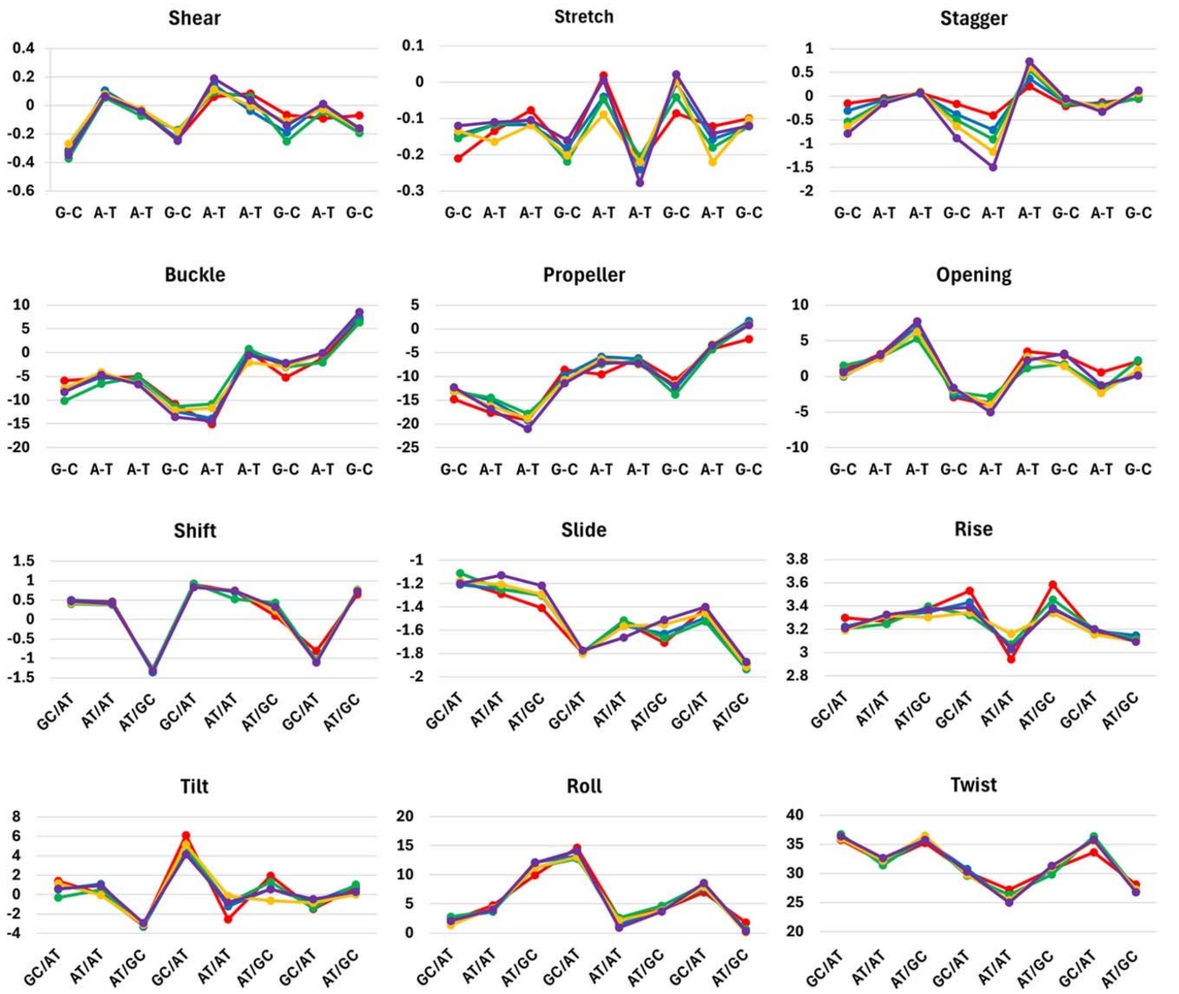

Figure S2 Helical parameters (intra- and inter-base pair helical parameters) in the Earth-1 (blue), in the Space-1 (green), in the Earth-2 (orange), and in the Space-2 (violet).

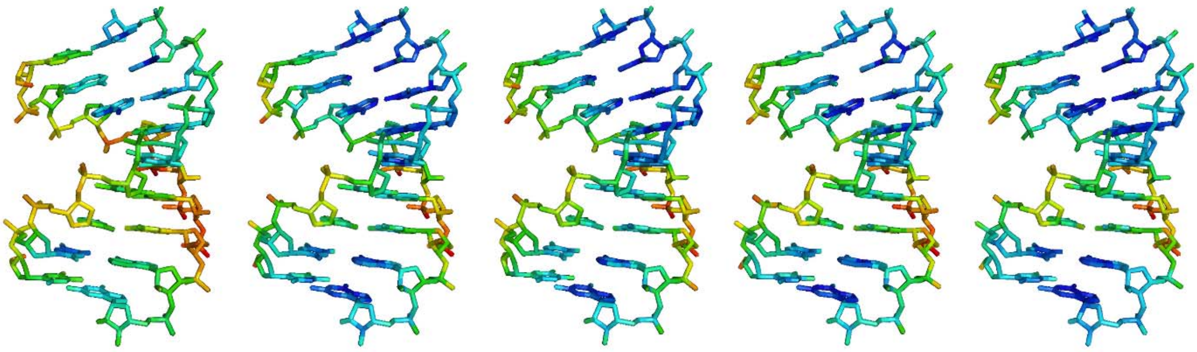

Figure S3 Overall structures colored to represent the temperature factor (B-factor). From left to right: previous study, Earth-1, Space-1, Earth-2, Space-2. The higher the B-factor, the warmer the color, and the lower the B-factor, the cooler the color. It can be seen that all four structures analyzed in this study have smaller B-factors than that in previous studies, especially at the ends of the molecules.
